# *In-situ* recording of ionic currents in projection neurons and Kenyon cells in the olfactory pathway of the honeybee

**DOI:** 10.1371/journal.pone.0191425

**Published:** 2018-01-19

**Authors:** Jan Kropf, Wolfgang Rössler

**Affiliations:** 1 Behavioral Physiology and Sociobiology (Zoology II), Biozentrum, University of Würzburg, Würzburg, Germany; 2 Centre for Neural Circuits and Behaviour, University of Oxford, Oxford, United Kingdom; University of California Santa Barbara, UNITED STATES

## Abstract

The honeybee olfactory pathway comprises an intriguing pattern of convergence and divergence: ~60.000 olfactory sensory neurons (OSN) convey olfactory information on ~900 projection neurons (PN) in the antennal lobe (AL). To transmit this information reliably, PNs employ relatively high spiking frequencies with complex patterns. PNs project via a dual olfactory pathway to the mushroom bodies (MB). This pathway comprises the medial (m-ALT) and the lateral antennal lobe tract (l-ALT). PNs from both tracts transmit information from a wide range of similar odors, but with distinct differences in coding properties. In the MBs, PNs form synapses with many Kenyon cells (KC) that encode odors in a spatially and temporally sparse way. The transformation from complex information coding to sparse coding is a well-known phenomenon in insect olfactory coding. Intrinsic neuronal properties as well as GABAergic inhibition are thought to contribute to this change in odor representation. In the present study, we identified intrinsic neuronal properties promoting coding differences between PNs and KCs using *in-situ* patch-clamp recordings in the intact brain. We found very prominent K^+^ currents in KCs clearly differing from the PN currents. This suggests that odor coding differences between PNs and KCs may be caused by differences in their specific ion channel properties. Comparison of ionic currents of m- and l-ALT PNs did not reveal any differences at a qualitative level.

## Introduction

Olfaction is a crucial sense for almost all animal species, and olfactory systems show striking similarities across a wide range of taxa—like the odorant receptor proteins or the glomerular neuronal architecture of the first relay station in the brain (for example reviewed by [1]). Honeybees need a powerful olfactory system for the location and evaluation of food sources, social (pheromone) communication, nestmate recognition, and for finding mating partners. Odorants are received by odorant receptors located in olfactory sensory neurons (OSNs) that are housed in different types of olfactory sensilla on the antennae (reviewed in [2]). OSN axons form two nerve bundles running along the antenna to the antennal lobe (AL) entrance and diverge into four distinct tracts (T1-T4) within the AL [3]. Here, OSN axons synapse on projection neurons (PNs) and local interneurons (LNs) forming spheroidal structures that are commonly termed olfactory glomeruli [2]. Glomeruli represent functional units of the AL and encode odor information in a spatially combinatorial fashion—theoretically allowing for coding an almost infinite number of odors (for example [4]). The exact synaptic connectivity in the honeybee AL between OSNs, LNs and PNs is not yet known. In the honeybee, three output tracts are formed by distinct groups of PNs: the medial (m-ALT), lateral (l-ALT) and medio-lateral antennal lobe tract (ml-ALT) [3,5,6]. They convey the olfactory information to the mushroom bodies (MBs) and the lateral horn (LH). The m-ALT is formed mainly by uniglomerular PNs and proceeds medially to the MB calyces and finally reaches the LH. The l-ALT, formed by another group of mainly uniglomerular PNs, runs just in the opposite direction and innervates the LH first before the axons target the MBs (reviewed in [7]). Both m- and l-ALT PNs possess axonal arborizations in the MBs and form distinct synaptic complexes, so called microglomeruli, with MB intrinsic neurons, the Kenyon cells (KC) [8,9]. The ml-ALT comprises multiglomerular PNs that do not innervate the MBs but project directly to the LH. Whereas multiple PN tracts can be found in various insect species including *Drosophila melanogaster* [10] and *Manduca sexta* [11], symmetrical dual olfactory pathways formed by uniglomerular PNs proceeding in parallel to the MBs, so far, have only been found in Hymenoptera [2,7,12]. The function of the dual olfactory pathway in parallel olfactory coding is still under debate. Both tracts convey information about a largely similar range of odorants [13–17] with only queen mandibular pheromone components and brood pheromone components as rare exceptions [18]. PNs of both tracts exhibit various response patterns, including tonic response patterns, phasic-tonic response patterns, bursts with a post burst hyperpolarization phase response patterns, and even inhibitory responses [5,13,17]. The reason for this general complexity in odorant response patterns may be that PNs need to convey a large amount of information from a very high number of OSNs (bottleneck). Approximately 60,000 OSNs [19] synapse on only ~900 PNs [20], which have to transmit the odor information reliably to a large number of KCs (~183,000 in total on each side of the brain) in the MBs [21,22].

In contrast to PNs, KCs are far more numerous and can be expected to employ less complex response dynamics. Indeed, calcium imaging experiments revealed that only a few honeybee KCs respond to the same odor. Furthermore, KC odor responses were shown to be shorter than both the actual odor stimulation and the PN odor response. KCs thus employ a temporally as well as spatially sparse information code [23]. The temporal response pattern of KCs was recently shown to be unaffected by GABAergic inhibition [24] and, therefore, can be assumed to be mainly caused by intrinsic electrical properties.

In the honeybee, intrinsic electrical properties of PNs and KCs, so far, have mostly been studied in primary cell cultures. PNs were shown to possess Na^+^, K^+^ and Ca^2+^ currents that are commonly known to be involved in action potential (AP) generation, and to contain Ca^2+^ dependent K^+^ channels, which could serve as an intrinsic self-inhibitory mechanism [25,26]. The whole-cell currents of KCs drastically differ from the ones found in PNs: KCs have very prominent A-type K^+^ currents [25,27–29] and most likely Ca^2+^ dependent K^+^ currents [30]. These were shown to be involved in sparse coding in cockroaches [31].

The aim of this study was to identify neuronal electrical properties supporting the complex odor response patterns of PNs [13] and the sparse coding properties of KCs [23,24] using *in-situ* patch-clamp recording techniques in the intact adult brain. Furthermore, using live tracing of PN cell bodies for proper identification of PN subtypes we were able to investigate, for the first time, m- and l-ALT PNs separately.

## Material and methods

### Animals

All honeybees (*Apis mellifera carnica*) were taken from colonies of our departmental bee station at the University of Würzburg. Only adult bees were used, but we did not control for the exact age of the bees. The bees were cooled in a refrigerator (4°C) and harnessed in custom-built plastic holders. Immediately after the bees had recovered from cooling, they were fed with a ~50% sucrose solution in distilled water.

### Pre-experimental PN staining

To distinguish PN cell bodies from LN cell bodies, we stained PNs 10 to 20 hours before recording. A small window was cut by removing the cuticle above the MB calyces, and glands and trachea covering the calyces were removed manually using fine forceps. Thin-walled glass micropipettes (1B100F-3, WPI, Sarasota, USA) were pulled with a Zeitz Puller (Zeitz-Instruments, Martinsried, Germany) and coated with Microruby^TM^ (tetramethylrhodamine dextran with biotin, 3,000 MW, lysine-fixable, D-7162; Molecular Probes, Eugene, Oregon, USA) dissolved (3–5%) in distilled water. The dye-coated micropipettes were used to puncture the calyces of both MBs, to have the dye subsequently taken up by the neurons. After the staining procedure, a two component tissue glue (Kwik-Sil^TM^ World Precision Instruments, Sarasota, USA) was used to seal the window and to prevent desiccation of the brain.

### Preparation for recording

All glands and trachea covering the brain were manually removed using fine forceps. Then, the heads were severed from the thorax. The head capsule was fixed in the recording chamber (RC-22C, Warner Instruments, LLC, Hamden, Connecticut, USA). With the head oriented in an upright fashion, this preparation allowed access to l-ALT PN and KC cell bodies. In order to gain access to m-ALT PN cell bodies, the complete preparation was turned upside down. The proboscis and the cuticle beneath the brain were removed using microscissors. Glands, muscles and tracheae covering the brain were gently removed. Additionally, the SEG was dislocated by pushing it into the direction of the MBs. To identify stained cell bodies, an Olympus imaging system (200×-800×, upright microscope: BX51WI, filter set: excitation 560/40 DCTX 590 emission 610 LP, objective: XLUMP, NA 0.95, light source: MT20, software: Cell R v2.5, all Olympus Imaging Europa, camera: model 8484-03G Hamamatsu Photonics) was used. The only neurons connecting the MBs with the AL are uniglomerular PNs–therefore, stained cell bodies in the AL could be clearly identified as l- and m-ALT PNs. KC cell bodies are numerous and can be easily identified. Differentiation between clawed (type II) and spiny (type I) KCs is possible according to their anatomical location. Only type II (clawed) KCs with cell bodies outside the calyx cup [22] were recorded in our experiments. This type of KCs is likely to receive convergent input from both PN tracts [7,32].

### Patch-clamp recordings

Patch-clamp electrodes were pulled from thick-walled borosilicate glass with filament (GB150F-8P, Science Products, Hofheim, Germany) with a Zeitz Puller. Electrodes had tip resistances of 4–6 MOhm (PN-recording) and 6–9 MOhm (KC-recording) measured in the extracellular solution. The patch-clamp electrodes were moved to the cell bodies by using an electrical micromanipulator (Junior Unit, Luigs & Neumann, Ratingen, Germany). For patch-clamp recordings, we used an Axopatch 200b amplifier, a Digidata 1440A acquisition board and the ClampEx software (all Molecular devices, Sunnyvale, California, USA). The extracellular solution comprised in mmol: NaCl (140), KCl (5), MgCl_2_ (1), CaCl_2_ (2.5), NaHCO_3_ (4), NaH_2_PO_4_ (1.2), HEPES (6) and glucose (14), adjusted to pH 7.4 with NaOH. The intracellular solution contained in mmol: K- gluconate (110), HEPES (25), KCl (10), MgCl_2_ (5), Mg-ATP (3), Na-GTP (0.5) and EGTA (0.5), pH 7.2. Currents were isolated by blocking Na^+^ currents and Na^+^ dependent currents with TTX (10^−7^ mol) and by blocking Ca^2+^ currents and Ca^2+^ dependent currents with CdCl_2_ (5x10^-5^ mol) in the extracellular solution. All chemicals were purchased at Sigma-Aldrich Chemie Gmbh (Munich, Germany) or at Carl Roth GmbH + Co. KG (Karlsruhe, Germany).

### Data analyses and statistics

Prior to voltage-clamp experiments, recordings were allowed to stabilize for approximately one minute. Voltage clamp protocols under standard conditions; TTX conditions and TTX/CdCl_2_ conditions were completed within 10 minutes after break-in. Leakage currents were subtracted using a p/5 protocol in ClampEx. The series resistance did not significantly change between recording under standard conditions and under TTX+ CdCl2 conditions in l-ALT PNs (paired Wilcoxon test: p>0.05). All patch clamp raw data traces were analyzed with the "statistics" function implemented in pClamp (Molecular devices, Sunnyvale, California, USA). The positive and negative maximal values at the transient peak and during the persistent phase were determined. Membrane voltages were directly measured with the digital meter on the amplifier and corrected by subtracting the liquid junction potential (13mV) of our solutions. Cell capacitances were obtained with the electrode test function implemented in ClampEx. The obtained data sets were further processed with Excel (Microsoft Corporation Redmond, Washington, USA), IV plots were also generated with Excel. All statistics as well as box plots were made with R (R Foundation for Statistical Computing, Vienna, Austria).

### Neuranatomical analyses and image composition

Brains stained with Microruby^TM^ were put into fixative solution (4% formaldehyde) overnight and rinsed five times for 10 min in PBS (phosphate-buffered saline, pH 7.2) the next day. Afterwards, the brains were dehydrated in an ascending ethanol series (50, 70, 90, 95, 2× 100%, each 10 min) and cleared in methyl salicylate (M2047, Sigma-Aldrich Chemie Gmbh, Munich, Germany). The brains were then mounted in methyl salicyate in custom made microscopy slides and scanned with a confocal laser-scanning microscope (Leica TCS SP2; Leica Microsystems, Wetzlar, Germany). Image stacks were processed with ImageJ 1.46j (Wayne Rasband, National Institutes of Health, Befesta, Md., USA). All images were finally arranged with CorelDrawX6 (Corel, Ottawa, ON, Canada).

## Results

### Pre-experimental identification of neurons

One important prerequisite for selectively recording from PNs was the identification of the respective neuronal cell bodies in the AL tissue, as they are embedded in many cell bodies of AL LNs [3]. The injection of dextran coupled dyes into the brain leads to the uptake of the dye in neurons with either pre- or post-synaptic profiles in the area where the dye was injected. The dye then is transported retrogradely as well as anterogradely along the respective neurites.

**Fig 1:**
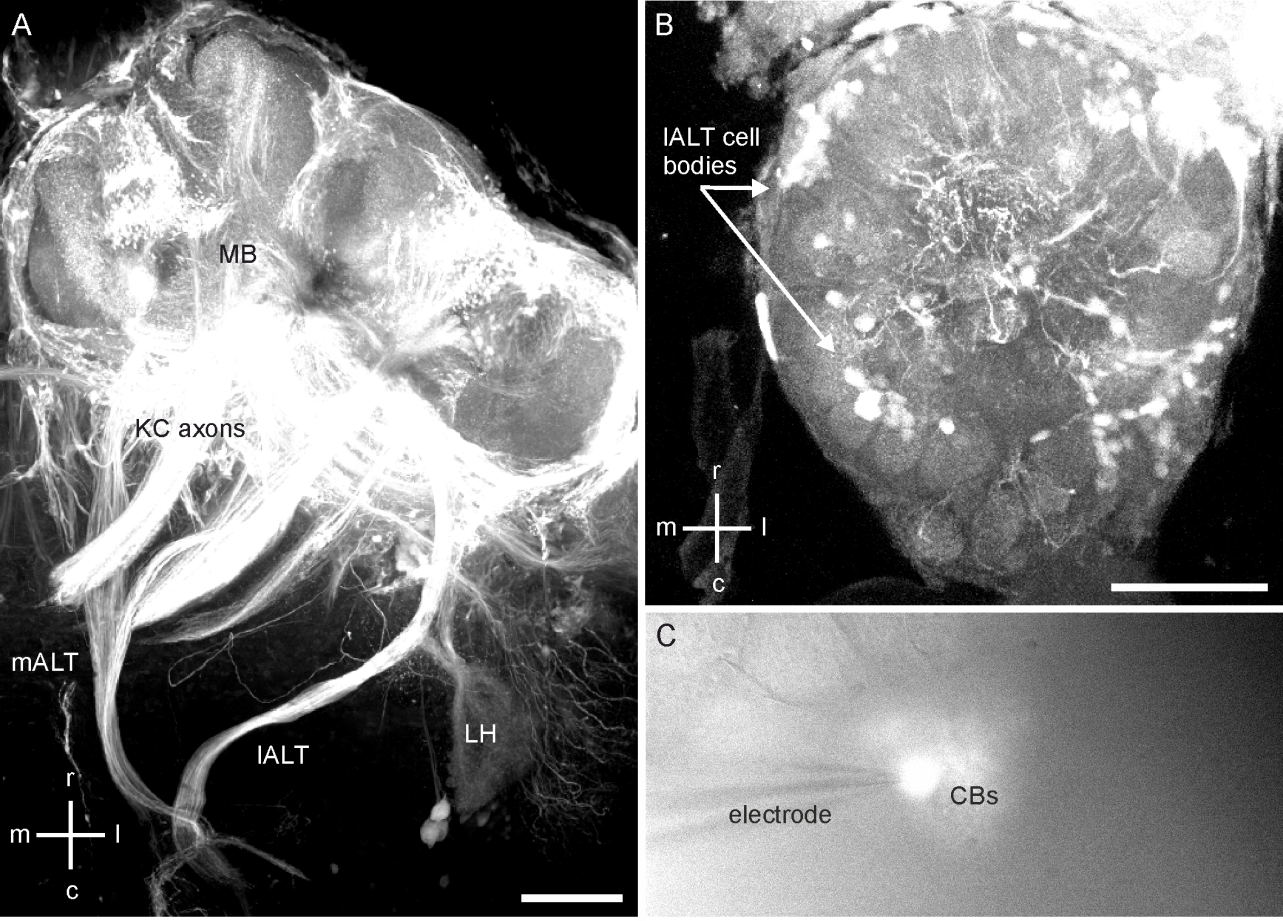
Pre-experimental identification of antennal lobe projection neurons. r = rostral, c = caudal, m = medial, l = lateral. A: Confocal microscopy stack showing the neurons stained in the mushroom body (MB) with Microruby^TM^ prior to *in situ* patch clamp recordings. The medial and the lateral antennal-lobe tract (m-ALT/l-ALT) of projection neurons as well as their arborizations in the lateral horn (LH) are clearly visible. Furthermore, Kenyon cell (KC) axons were stained. Bar 100 μm. B: Substack of the ventral part of the antennal lobe (AL). Cell bodies from l-ALT neurons and their glomerular branching patterns are clearly visible. Bar 100 μm. C: A cluster of stained cell bodies (CBs) viewed with a fluorescence microscope. Only stained cell bodies were used for PN recordings. The patch-clamp electrode is attached to a stained l-ALT projection neuron cell body.

Confocal microscopy analyses after the injection of Microruby^TM^ into the MBs revealed that the dye was taken up by KCs, PNs from the optic ganglia, PNs from the subesophageal (SEG) tract (potentially gustatory and/or mechanosensory PNs), and olfactory PNs from the antennal lobe. In a broad overview, KCs proceeding from the MB calyces to the vertical lobe, visual commissures connecting the optic lobes, and tracts from SEG PNs and olfactory PNs ascending from the AL to the MBs could be distinguished (Fig 1A). However, the only cells with axonal arborizations in the MBs as well as cell bodies in the AL are uniglomerular olfactory PNs [3]. Therefore, cell bodies in the AL stained after dye injection into the MBs could clearly be identified as PNs ascending to the MBs. PN ramifications within glomeruli as well as stained cell bodies clustered around the AL were visible and can be identified as l- and m-PNs according to anatomical data from Fig 2 in [3] (Fig 1B). We approached these PN cell bodies *in situ* using a fluorescence light microscope (Fig 1C).

### Recording under standard conditions

As this represents the first study that comparatively investigated the composition of ionic currents of honeybee PNs as well as KCs by *in situ* recordings in the intact brain, we started by analyzing and comparing basic neuronal properties of the three neuronal classes under standard conditions. The membrane voltages of l-ALT PNs under our recording conditions ranged from -30 to -50mV–the mean membrane voltage of all cells was -42.0 mV (N = 12). In contrast, KC membrane voltages were more negative and ranged from -42 to -61 mV (mean = -52.0 mV, N = 7). As the whole-cell patch clamp technique can induce leakage currents, which potentially depolarize the cells, we assumed even lower membrane voltages in intact cells. Therefore, during current clamp recordings, a minimal negative current was injected to achieve resting potentials of -60 mV in PNs and -80mV in KCs.

**Fig 2:**
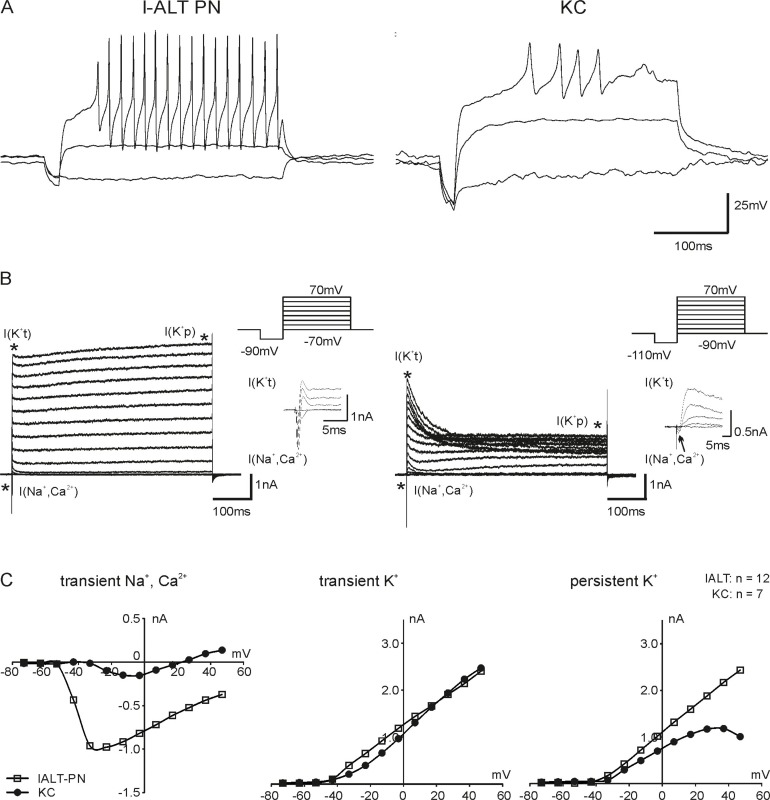
A: Action potentials (AP) of a honeybee lateral antennal-lobe tract projection (l-ALT PN) and a Kenyon cell (KC) elicited in current-clamp mode. The PN started to generate APs at ~ -50mV, the KC at -40 mV. B: Representative voltage clamp recordings of an l-ALT PN and a KC. L-ALT and m-ALT PNs have similar currents, but KCs differ drastically as they have only small I(Na^+^,Ca^2+^) and a prominent I(K^+^t) with about twice the size compared to the I(K^+^p). PNs were kept at -70 mV, KCs at -90 mV. In both PNs and KCs, a quick hyperpolarizing step of -20 mV was used, then the membrane voltage was increased in 10mV increments. C: I-V plots of the mean currents of the two neuronal types. Note the persistent K^+^ current decreasing above 30 mV hinting Ca^2+^ dependent K^+^ currents in the I(K^+^p) in KCs.

Because l-ALT PN cell bodies are accessible without turning the preparation around and removing parts of the SEG l-ALT PNs recordings proved to be much easier than recording from m-ALT PNs. Therefore, we first focused on l-ALT PN recordings for the comparison between PNs and KCs. PNs recorded in current clamp started to fire action potentials at membrane voltages from -50 to -40 mV, and KCs started to fire action potentials at -30 mV (Fig 2A). In voltage-clamp recordings under standard conditions, all recorded neurons showed transient inward, transient outward and persistent outward currents (Fig 2B). Considering the ion concentrations we used for recording under standard conditions, we conclude that the inward currents were mainly Na^+^ currents and only partly Ca^2+^ currents, whereas all outward currents likely were K^+^ currents. The currents that were activated first during the voltage step protocol (-70 to 70 mV in PNs, -90 to 70mV in KCs) were inward currents. The inward currents of PNs were activated at approximately -50 mV, the inward currents of KCs at -30 mV (Fig 2B). The transient K^+^ currents were activated right after the inward currents at -40 mV in both PNs and KCs (Fig 2B). These currents deactivated very fast in PNs, and they were only slightly higher than the persistent K^+^ currents. In contrast, KCs exhibited a very prominent transient outward current with about twice the amplitude of the persistent K^+^ current. This current activated and deactivated significantly slower than the transient current in PNs (Fig 2B). Persistent currents activated at -30mV in both neuron types, yet they showed different IV relations in PNs and KCs. PN persistent currents increased with increasing membrane voltages in a linear IV relationship (Fig 2C). Conversely, the persistent K^+^ current of KCs showed a nonlinear, distinct N-shape in the IV plot, which increased until 30 mV to then decrease until reaching 50 mV (Fig 2C). This N-shape hints at the presence of Ca^2+^-dependent K^+^ currents, which decrease once the driving force for Ca^2+^ declines (for example, [31]).

### l-ALT PN currents under the influence of TTX and CdCl_2_

**Fig 3:**
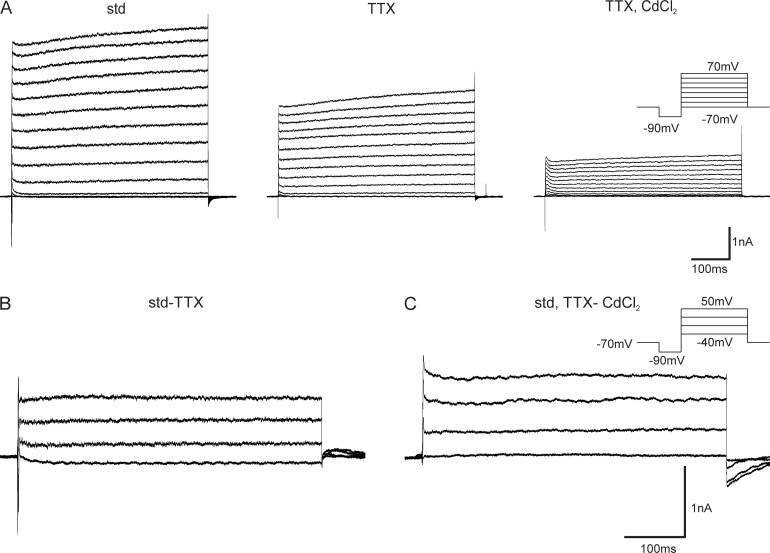
Pharmacological blocking of Na^+^, Ca^2+^, Na^+^ dependent and of Ca^2+^ dependent currents in lateral antennal-lobe tract (l-ALT) projection neurons (PN). A: Representative voltage clamp recordings of l-ALT PNs under standard conditions (std), TTX and TTX / CdCl_2_ conditions. Most of the transient inward current was already blocked by TTX, the outward current was also partly blocked. All inward currents were blocked under TTX / CdCl_2_ conditions, and large parts of the outward currents are blocked, too. B: TTX (std-TTX) sensitive currents were isolated by subtracting the current trace obtained under TTX conditions from the current trace obtained under standard conditions. C: CdCl_2_ (std,TTX-CdCl_2_) sensitive currents were isolated by subtracting the current trace obtained under TTX + CdCl_2_ conditions from the current trace obtained under standard + TTX conditions.

**Fig 4:**
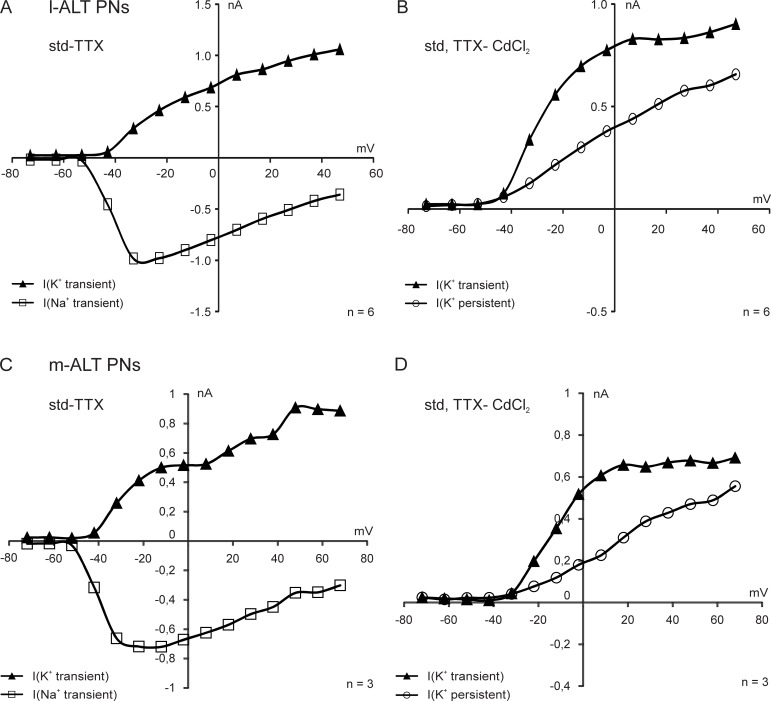
Comparison after pharmacological blocking of Na^+^, Ca^2+^, Na^+^ dependent and Ca^2+^ dependent currents in lateral and medial antennal-lobe tract projection neurons (l-ALT PNs, m-ALT PNs). A, B: Mean current-voltage plots of TTX sensitive (A) and CdCl_2_ sensitive (B) isolated currents in l-ALT PNs Transient Na^+^ and K^+^ currents as well as persistent K^+^ currents can be observed. C, D: Mean current-voltage plots of TTX sensitive (C) and CdCl_2_ sensitive (D) isolated currents in m-ALT PNs. Transient Na^+^ and K^+^ currents as well as persistent K^+^ currents can be observed.

As the IV relationships we observed under standard conditions indicated Na^+^ currents, K^+^ currents as well as Ca^2+^ sensitive K^+^ currents (Fig 2B and 2C), we used tetrodotoxin (TTX) and CdCl_2_ as primary pharmacological agents. TTX (10^−7^ mol), a toxin which is well known from pufferfish, blocks voltage dependent Na^+^ channels and Na^+^ dependent K^+^ channels. CdCl_2_ (5x10^-5^ mol) blocks Ca^2+^ channels and, therefore, also affects Ca^2+^ dependent K^+^ channels. In the IV plots obtained after adding TTX to the extracellular solution, the fast inward currents were almost completely abolished in PNs (Fig 3A). Thus, we conclude that the inward currents are dominated by Na^+^ currents. Additionally, both the transient and the persistent K^+^ current were strongly reduced after adding TTX, which leads to the conclusion that the outward current partly consists of Na^+^ dependent K^+^ currents (K_Na_). To be able to identify purely TTX sensitive currents, we subtracted the IV curves obtained with TTX in the extracellular solution as a control from the curves obtained under standard conditions (Fig 3B). The purely TTX sensitive traces revealed a very fast voltage dependent Na^+^ current, a very fast peak K_Na_ and a sustained K_Na_. The two TTX sensitive K^+^ currents, the very fast transient, and the persistent K^+^ current are all K_Na_ currents activated by the fast Na^+^ inwards current and the sustained Na^+^. The transient K_Na_ current activated at a slightly higher membrane voltage than the Na^+^ inward current and increased drastically until 0 mV. It increased only marginally above 0 mV. (Fig 4A). In a further step, CdCl_2_ was added to the extracellular solution to block all Ca^2+^ currents and, therefore, also the K^+^ currents depending on extracellular Ca^2+^ (K_Ca_). These currents were analyzed by subtracting the IV traces obtained after adding CdCl_2_ to the extracellular solution from the traces obtained under standard + TTX conditions (Fig 3C). The current subtraction revealed a slight peak of inward currents between -30 and -20 mV indicating the presence of Ca^2+^ currents. Furthermore, two K_Ca_ currents, a transient current (K_Ca t_) and a persistent current (K_Ca p_), could be observed. Both K_Ca t_ current and K_Ca p_ current activated between -40 and -30 mV. The K_Ca t_ current increased until 0 mV and reached a plateau there, which is, similar to the decreasing transient K_Na_, most likely due to a decreasing driving force for Ca^2+^ ions. Again, K_Ca p_ did not reach plateau levels (Fig 4B).

In summary, l-ALT PNs exhibit transient Na^+^ currents (Na_t_), transient Na^+^ dependent K^+^ currents (K_Na t_)), Ca^2+^ currents, transient Ca^2+^ dependent K^+^ currents (K_Ca t_), persistent Ca^2+^ dependent K^+^ currents (K_Ca p_).

### Qualitative comparison between m-ALT and l-ALT PNs

In a set of pilot experiments, we selectively recorded from m-ALT PNs using the head preparation with fluorescently labeled PNs in an upside down position and after removal of the SEG. Recording of m-ALT PNs, therefore, was much more difficult compared to l-ALT PN recordings. The mean resting membrane voltage of m-ALT PNs was -39.4 mV (N = 4) and close to the l-ALT PN resting membrane voltage -42.0 mV (N = 12). Pharmacological isolation of currents did not reveal any qualitative differences between m- and l-ALT PNs (Fig 4). M-ALT PNs also exhibit transient Na^+^ currents (Na_t_), transient Na^+^ dependent K^+^ currents (K_Na t_), Ca^2+^ currents, transient Ca^2+^ dependent K^+^ currents (K_Ca t_), and persistent Ca^2+^ dependent K^+^ currents (K_Ca p_) (Fig 4C and 4D). Although, due to the difficulties of the upside down preparation, recordings in this experiment could only be obtained from a limited number of m-ALT PNs, the results, at a qualitative level, indicate a high degree of similarity in the electrical properties of l-ALT and m-ALT PNs under standard conditions.

### Comparison of transient currents in PNs and KCs

**Fig 5:**
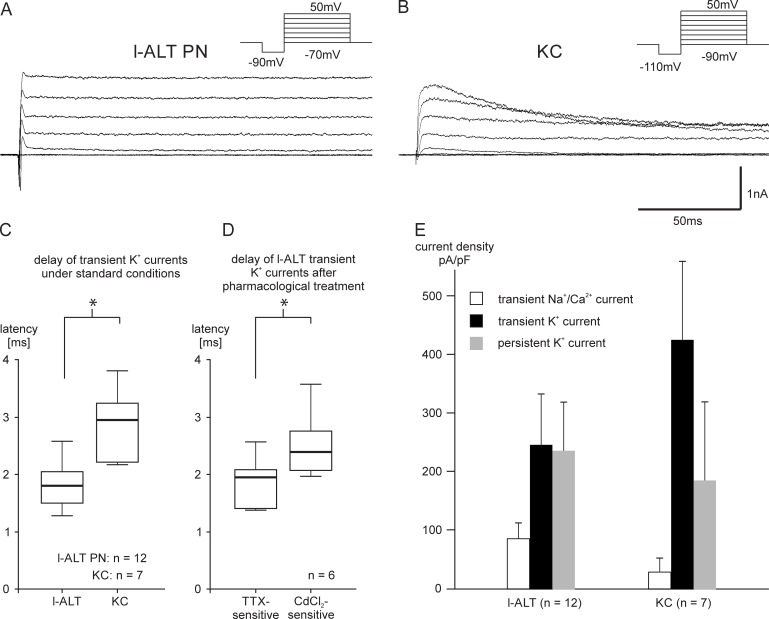
Transient current analyses. A, B: Representative voltage clamp recordings from a lateral antennal-lobe tract projection neuron (l-ALT PN) (A) and a Kenyon cell (KC) (B). C: Delay of the transient K^+^ currents. The delay was significantly shorter in l-ALT PNs compared to KCs (p<0.05). D: Delay of l-ALT TTX and CdCl_2_ sensitive transient K+ currents (p<0.05). E: Mean current densities of l-ALT and KCs. Note the high current density of the transient K^+^ current and the low current densities of the Na^+^, Ca^2+^ currents in KCs.

The transient K^+^ currents very clearly differed between PNs and KCs. Our pharmacological analysis revealed that PN transient K^+^ currents are mostly dependent on Na^+^ or Ca^2+^ influx (Fig 3). Furthermore, in contrast to the persistent K^+^ current, the transient K^+^ current in KCs rose linearly and, therefore, hints at a mostly voltage-dependent transient K^+^ current (Fig 2C). By looking at an expanded time scale, it becomes obvious that PN transient K^+^ currents activate and deactivate faster than KC transient K^+^ currents (Fig 5A and 5B). To analyze the timing of the activation of the transient K^+^ current, we determined its latency, which is significantly shorter in l-ALT PNs than in KCs (Fig 5C, Wilcoxon test l-ALT vs KC: p<0.05). As the transient K^+^ currents are likely to be Na^+^ or Ca^2+^ dependent, we also analyzed the latency of the transient currents after pharmacological blocking (Fig 5D). The TTX-sensitive transient K^+^ current was significantly faster than CdCl_2_-sensitive transient K^+^ currents (TTX vs CdCl_2_: p<0.05). In addition to the different timing between PN and KC transient K^+^ currents, there are also differences in its maximal amplitude. Generally, PNs are larger than KCs (mean cell capacitance m-ALT: 17.27 pF, N = 4, l-ALT; 13.12 pF, N = 12; KC: 6.18 pF, N = 7). To adjust for the differences in their cell size, we calculated the current densities of PNs and KCs (Fig 5E). The transient Na^+^/Ca^2+^ currents of KCs are especially small (mean maximal current: 44pA) whereas the transient K^+^ current is relatively large (mean maximal current: 1192pA) (Fig 5E).

## Discussion

We performed, for the first time, selective *in situ* whole whole-cell voltage clamp recordings from l- and m-ALT PNs and from KCs in the intact brain to compare their electrical properties. The differences between KCs and PNs were prominent: PNs possess strong transient Na^+^ currents as well as Na^+^ dependent transient K^+^ currents and Ca^2+^ dependent transient and persistent K^+^ currents. KCs, in contrast, have a relatively small transient Na^+^ current, a prominent transient K^+^ current, and a most likely Ca^2+^ dependent persistent N-shaped K^+^ current.

### Electrophysiological properties of PNs

In our recordings, PNs fired action potentials starting at membrane voltages of around -40 mV without any spike frequency adaptation. PNs had cell capacitances of about 13–17 pF and a mean membrane resting potential of around -40 mV. The capacitances indicate that the cells measured *in situ* were slightly larger than cultured PNs that were reported around 11 pF [25]. This could be due to the fact that neurons were primary cultures from pupal stages. Although the PNs in primary cultures did grow dendritic processes, we assume that the difference at least partly is due to substantially larger dendritic and axonal arborizations in *in situ* measured PNs compared to cultured neurons. A resting membrane potential of -40 mV appears rather depolarized. We assume that the resting potential of PNs approximately ranges between -50 and -60 mV, which resembles the lowest measured resting potentials. This may be due to damage either during the preparation, staining or rupturing of the membrane patch. No significant differences between m- and l-ALT PNs regarding their capacitances or their resting membrane potentials were found.

PNs possess prominent Na^+^ currents, K_Na_, and K_Ca_ currents. The IV relations observed under standard conditions partly resemble the ones recorded in primary cell cultures [25,26]. In both *in vitro* studies and in our *in-situ* study, PNs exhibit a fast transient Na^+^ current, a fast activating and deactivating transient K^+^ current and a persistent K^+^ current. However, Grünewald [25] described a non-linear IV relation for the persistent K^+^ current leading to an N-shape of the IV plot for cultured PNs. Perk and Mercer [26] recorded two types of cultured AL neurons, and only one of the two types displayed IV relationships similar to our recordings. The other type did not display a fast transient K^+^ current, but instead had a non-linear N-shaped persistent K^+^ current. We conclude from our recordings that the first type measured by Perk and Mercer [26] was a PN type, whereas the second type may be a LN type. The only explanation for the differences between our recordings and the primary cell culture PN recordings from Grünewald [25] are differences between *in situ* (adult) and *in vitro* (pupal phase) ion channel expression.

The fast Na^+^ current resembles the typical Na^+^ current necessary for fast depolarization during action potential generation. This current was sensitive to TTX, which blocks a wide range of Na^+^ channels and prohibits action potential generation.

We observed a fast transient K_Na_ current that activated at -40 mV and increased until -10 mV to reach a maximal current plateau. Additionally, the plateau value was reached at the voltage level when the Na^+^ current declined. Concerning their timing, K_Na t_ currents seem to be the fastest transient K^+^ currents in PNs. K_Na t_ currents have been described in DUM neurons in cockroaches [33] and are thought to contribute to the limitation of action potential duration [34].

In our study, Ca^2+^ currents were measured after blocking Na^+^ currents with TTX. As K^+^ currents were not additionally blocked, the full magnitude of the Ca^2+^ currents could not be investigated in our studies. Nevertheless, the results strongly indicate that honeybee AL PNs possess a transient Ca^2+^ current, which appeared at -30 to -20 mV. To precisely determine activation thresholds and dynamics of Ca^2+^ currents, further experiments are necessary.

In addition to the K_Na_ current, we observed a transient and a persistent K_Ca_ current. Both K_Ca_ currents seem to be not purely Ca^2+^ dependent, but also voltage dependent. The activation membrane voltage, the existence of a transient and a persistent component, as well as the plateau reached by the transient current together with the linear IV relation of the persistent component are all properties of the pSlo subunit of the BK channel described in cockroach DUM neurons [35] and also cockroach PNs [36]. A similar current was observed in cultured AL neurons recorded by Perk and Mercer [26]. However, in cultured honeybee PN recordings from Grünewald [25] no such current was observed. Instead, the K_Ca_ current exhibited a descriptive N-shape in the IV plot [25], which is typical for SK K_Ca_ currents that are purely Ca^2+^ dependent and not voltage-dependent (reviewed by [37]). We did not observe an N-shape of the K^+^ current. Apart from the general difference to the *in-situ* situation and potential developmental aspects, we have no comprehensive explanation for these differences with recordings of cell cultured PNs by Grünewald [25]. To finally determine the nature of the K_Ca_ channels, specific pharmacological blocking with Apamin, a selective SK channel blocker, and Iberiotoxin, a selective BK channel blocker [38], is necessary. Transient K^+^ currents influence AP shape, the total AP duration [39], and the magnitude of the post AP hyperpolarization [35].

Based on our qualitative comparison with a limited number of m-ALT PNs, we assume that the ionic current properties do not strongly differ from l-ALT PNs (Fig 5). However, for a more comprehensive understanding of the ionic currents in both types of neurons, further pharmacological experiments are necessary. To be able to do this under *in-situ* conditions, further improvements of the preparation and recording techniques are necessary.

### Electrophysiological properties of KCs

KCs had mean membrane resting potentials between -42 and -61 mV, and a mean capacitance of 6.18 pF. Similar as in PNs, we suppose that membrane potentials may be lower in un-manipulated neurons, and we expect membrane potentials of -60 to -65mV, which would be consistent with the mean potential (-62.1 mV) in *in situ* recordings of honeybee KCs in isolated brains [30]. The capacitance measured by Palmer et al. [30] amounted 3.2 pf. In current clamp, KCs showed spike frequency adaptation after pronounced depolarization, which was also observed by Palmer et al. [30].

Under standard conditions, KCs exhibited a fast inward current, which is likely to be a Na^+^ current. The outward currents can roughly be divided into a transient, A-shaped K^+^ current and a persistent current with a non-linear IV relation leading to an N-shaped IV plot. The transient K^+^ current activates at -45 mV, the Na^+^ and the persistent K^+^ current activate at ~ -40 mV. The activation voltages of the currents as well as the basic shape of the IV plots are consistent with the IV plots obtained from KCs in cell culture [25,27,28] and recordings in isolated brain preparations [30]. The only prominent difference is that no N-shape was observed in cell cultured KCs [25]. The most prominent current observed in KC recordings was the transient K^+^ current. This current activated at ~ -45 mV and increased linearly in a voltage dependent manner. In a single recording, it remained almost unaffected by blocking of Na^+^ channels with TTX and blocking of Ca^2+^ channels with CdCl_2_. This points towards an A-type K^+^ current. Additionally, its activation threshold and dynamics are similar to A-type K^+^ currents in all studies on primary KC cultures [25,27,28].

The N-shape of the persistent K^+^ current is normally caused by K_Ca_ currents. The Ca^2+^ influx after depolarization diminishes once the driving force for Ca^2+^ ions is reduced. Therefore, the K_Ca_ current will also decrease leading to an N-shaped IV plot. The typical shape was reported both in honeybee KC primary cultures [27] and in the isolated brain preparation [30] but was not found in another cell-culture patch clamp study. Similar K_Ca_ currents were found in cockroaches, yet their amplitude was about twice as large as the amplitude of the A-type K^+^ current [31].

### Potential influence of ionic currents on odor coding

The subset of currents we found in PNs is relatively similar to currents observed in cockroach DUM neurons (reviewed by [34]). Especially K_Ca_ currents closely resembling K_Ca_ currents recorded in our study were shown to affect action potential waveform and excitability [36,39]. These currents allow PNs to be well suited as neurons with relatively high response probabilities and spontaneous AP frequencies producing reliable responses to synaptic input. On a qualitative level, we did not find any obvious differences between the currents of l- and m-ALT PNs. This may indicate that coding differences between PNs of the two tracts in terms of spontaneous activity and odor response frequencies [17,32] most likely are caused by differences in synaptic connectivity between OSNs, LNs and PNs. In addition, differences in synaptic currents may contribute to coding differences. For example, OSNs of a specific type of olfactory sensilla (*Sensillum basiconicum*) preferentially project to m-ALT associated glomeruli [40] indicating that the OSN-PN connectivity varies between the two sets of PNs. Furthermore, differences in LN connectivity between the two AL hemilobes may as well affect odor coding in m- and l-ALT PNs [41].

KCs do have a completely different set of ionic currents than PNs. Instead of the very fast transient K_Ca_ and K_Na_, KCs have slower transient A-type and persistent N-shaped K^+^ currents similar to cockroach KCs [31]. Generally, KCs are thought to play a role in coincidence detection and express synaptic plasticity [7,32,42–44]. For coincidence detection and spatial separation of information, neurons need to encode information in a spatially and temporally sparse fashion. Calcium-imaging studies support this temporal sparseness in KCs [23,24], even though responses in presynaptic PN axonal boutons outlasted the entire odor stimulus [23]. Such a drastic change in stimulus representation can be caused by strong transient K^+^ currents in combination with K_Ca_ currents or by GABAergic inhibition. Honeybee KCs do receive GABAergic input in the MB calyx [45,46], which could lead to self-inhibition after odor stimulation [47], yet Froese et al. [24] show that blocking of the fast ionotropic part of the GABA response did not affect the fast termination of odor responses in KCs. Therefore, it is more likely that ionic currents cause the change in stimulus representation, as shown in the cockroach [31]. Furthermore, SK channels in *Manduca* PNs are thought to mediate an after hyperpolarization following pheromone responses [48,49]. Both the N-shaped K_Ca_ currents in cockroaches (31) and the SK channels in *Manduca* PNs [48,49] are likely to shorten odor responses. Both the transient and the persistent K^+^ currents we observed in honeybee KCs are relatively large compared to the inward currents and can therefore support the generation of temporally sparse, phasic responses. Whether the dominant transient K^+^ currents or the persistent K_Ca_ currents are more important for temporally sparse coding remains to be investigated. In addition to the temporal sparseness of stimulus representation, the number of excited KCs (spatial sparseness) responding to a given stimulus is also important. In *Drosophila*, blocking of GABAergic feedback neurons in the MBs resulted in activation of higher KC numbers (less spatial sparseness) [50]. Considering both morphological and physiological features of these neurons, we propose that feedback neurons inhibiting KCs most likely promote spatial sparseness in the representation of odor stimuli in the MBs, whereas the temporal sparseness may be caused by intrinsic KC neuronal properties.
